# Changes in self-reported HIV testing during South Africa's 2010/2011 national testing campaign: gains and shortfalls

**DOI:** 10.7448/IAS.19.1.20658

**Published:** 2016-04-11

**Authors:** Brendan Maughan-Brown, Neil Lloyd, Jacob Bor, Atheendar S Venkataramani

**Affiliations:** 1 Southern Africa Labour and Development Research Unit, University of Cape Town, Cape Town, South Africa; 2 Department of Global Health, Boston University School of Public Health Boston, MA, USA; 3 Division of General Internal Medicine, Massachusetts General Hospital, Harvard Medical School, Boston, MA, USA; 4 Center for Population and Development Studies, Harvard University, Cambridge, MA, USA

**Keywords:** HIV/AIDS, testing, HCT, southern Africa, socio-economic determinants, disparities

## Abstract

**Objectives:**

HIV counselling and testing is critical to HIV prevention and treatment efforts. Mass campaigns may be an effective strategy to increase HIV testing in countries with generalized HIV epidemics. We assessed the self-reported uptake of HIV testing among individuals who had never previously tested for HIV, particularly those in high-risk populations, during the period of a national, multisector testing campaign in South Africa (April 2010 and June 2011).

**Design:**

This study was a prospective cohort study.

**Methods:**

We analyzed data from two waves (2010/2011, *n*=16,893; 2012, *n*=18,707) of the National Income Dynamics Study, a nationally representative cohort that enabled prospective identification of first-time testers. We quantified the number of adults (15 years and older) testing for the first time nationally. To assess whether the campaign reached previously underserved populations, we examined changes in HIV testing coverage by age, gender, race and province sub-groups. We also estimated multivariable logistic regression models to identify socio-economic and demographic predictors of first-time testing.

**Results:**

Overall, the proportion of adults ever tested for HIV increased from 43.7% (95% confidence interval (CI): 41.48, 45.96) to 65.2% (95% CI: 63.28, 67.10) over the study period, with approximately 7.6 million (95% CI: 6,387,910; 8,782,986) first-time testers. Among black South Africans, the country's highest HIV prevalence sub-group, HIV testing coverage improved among poorer and healthier individuals, thus reducing gradients in testing by wealth and health. In contrast, HIV testing coverage remained lower for men, younger individuals and the less educated, indicating persistent if not widening disparities by gender, age and education. Large geographic disparities in coverage also remained as of 2012.

**Conclusions:**

Mass provision of HIV testing services can be effective in increasing population coverage of HIV testing. The geographic and socio-economic disparities in programme impacts can help guide best practices for future efforts. These efforts should focus on hard-to-reach populations, including men and less-educated individuals.

## Introduction

HIV counselling and testing (HCT) is a crucial component of global HIV prevention and treatment efforts. HIV testing is the entry point for antiretroviral therapy, which reduces AIDS-related morbidity and mortality and increases life expectancy [1,2]. Identifying HIV-positive persons early in their disease progression, while they are still asymptomatic, is critical for reaping the full therapeutic and preventive benefits of antiretroviral treatment [3,4]. At the population level, HIV testing is the bedrock of the UNAIDS 90-90-90 strategy [5], which seeks to diagnose 90% of individuals living with HIV, link 90% of those individuals to treatment and achieve viral load suppression in 90% of those individuals by 2020, to prevent further transmission of the disease [3,6,7].

However, current testing rates lag far behind these targets [8]. Consequently, large-scale testing campaigns have been touted as a means to achieve them [9]. In April 2010, the government of South Africa – the site of the world's largest HIV epidemic [8] – launched “a massive campaign to mobilize all South Africans to get tested for HIV and to ensure that every South African knows their HIV status” [10]. This nationwide, multisector campaign targeted all individuals for HCT, with special emphasis placed on men, sexually active individuals aged 15 to 49 years, pregnant women and marginalized populations [10].

At the national level, campaign activities included demand creation, including promotion of HCT via mass media (across television and radio in all 11 official languages); policy mandates, specifically around the introduction of opt-out provider-initiated HCT to all clients attending a healthcare facility; and provision of general guidance and support around mobilizing HCT providers and scaling-up diverse modalities such as mobile clinics and home-based HCT [11]. However, the specifics of programme activities and implementation strategies were left to the discretion of districts. Although information on exactly what activities each district engaged in is not available, government reports suggest that these included (1) a spectrum of social mobilization interventions, such as door-to-door distribution of pamphlets; (2) provision of HCT information and services at mass events (e.g. sports and religious); and (3) mobilization and engagement of key populations such as youth, traditional leaders, religious groups, business leaders and employees. Districts were also placed in charge of data collection and monitoring and evaluation [10].

According to government reports, the campaign was a success, with over 20 million HIV tests conducted during the campaign period [12–14]. These aggregate statistics, however, do not shed light on (1) *the extent to which the campaign reached previously untested individuals* and (2) *whether the campaign was successful in ameliorating population disparities in testing*, which was one of its stated goals [11]. The literature on HIV testing in sub-Saharan Africa documents disparities in HIV testing by gender, age, education, employment status and wealth [15–25]. These disparities are important given their relationship with HIV risk – for example, less-educated individuals are less likely to test for HIV but are at higher risk of infection [26,27] – and for what they portend: rising disparities in who benefits from HIV care services. Understanding the impacts of HIV testing campaigns on first-time testing, particularly among vulnerable populations, is of particular importance as countries begin to scale up treatment-based prevention efforts.

Consequently, our study sought to answer two main questions. First, how many individuals tested for the first time during the South African campaign? Second, was the programme effective in overcoming persistent geographic, demographic and socio-economic disparities in HIV testing uptake, particularly among groups at highest risk of contracting HIV?

## Methods

### Data and measures

We used individual-level data on persons aged 15 and older from the National Income Dynamics Study (NIDS), a nationally representative cohort study. This study was reviewed and approved by the ethical review committee of the University of Cape Town. The first three waves of NIDS were conducted in 2008, 2010/2011 and 2012. We used data from the second (2010/2011) and third (2012) waves, which fielded questions about HIV testing (Wave 1 did not). Wave 2 thus served as our baseline.

The initial NIDS sample was drawn using a two-stage design consisting of a random selection of primary sampling units (PSUs), stratified over South Africa's 53 districts, and a random sample of dwelling units from each PSU. In Waves 2 and 3, questionnaires were completed by 16,893 and 18,707 individuals 15 years and older. Attrition between Waves 2 and 3 was 17.3% [28].

Our primary outcome measure, having ever tested for HIV, was collected with the following question, fielded in 2010/2011 and 2012: “I do not want to know the result, but have you ever had an HIV test?” Possible response options included “yes,” “no,” “don't know” and “refuse [to answer].” We created a binary variable equal to 1 for individuals who reported having been tested for HIV and 0 for individuals answering otherwise.

We examined demographic data on age, gender, religiosity, marital status and race (a categorical variable distinguishing between white, black African, Indian/Asian and coloured individuals; “coloured” is a common and socially acceptable term in South Africa for individuals of mixed race). For geography, we considered the province of residence and distinguished between rural and urban areas. Socio-economic characteristics included household per capita income, schooling (a continuous variable ranging from 0 to 18 years) and employment status.

Given previous studies indicating that a large proportion of people living with HIV present for HIV testing only after they have become very sick [29], we also considered health characteristics. Specifically, we measured self-reported health status at the time of the survey (a five-point Likert scale denoting states of health ranging from “poor” to “excellent”). Given that poor mental health and substance use may act as barriers to health service utilization [30,31], we also included a measure of depression (based on the Center for Epidemiologic Studies Depression Scale) [32] and a binary indicator of alcohol consumption. Last, we created an indicator of ever being pregnant between our surveys, as women routinely receive HIV testing from antenatal clinics (see Supplementary Table 1 for details on all measures).

### Analysis

We first computed descriptive statistics for the baseline 2010/2011 survey. We then estimated the number of first-time testers during the study period by assessing differences in the proportion of individuals ever tested for HIV between the 2010/2011 and 2012 surveys. It is important to note that the 2010/2011 data collection coincided with the campaign and, consequently, we may be underestimating the number of new testers (if some respondents who reported ever having tested for HIV were actually first tested as part of campaign efforts). It is also possible that some portion of the change in the percentage ever tested could be driven by individuals testing for the first time before our 2012 survey, but after the end of the HCT campaign, which could bias upwards estimates of first-time testers. Furthermore, some individuals would have tested even in the absence of a mass national HCT campaign, due to pre-existing trends in testing. To address this last possibility we examined data on the number of HIV tests conducted nationwide each month from the Department of Health both before and during the national testing campaign.

We then assessed whether the national campaign ameliorated disparities in testing by conducting a number of descriptive and regression sub-group analyses. We focused on differential HIV testing uptake among several sub-populations within the black African population, among whom HIV prevalence is the highest [8]. Specifically, we examined the proportion of black African men and women ever tested for HIV in 2010/2011 and 2012 separately by age, education, income (above and below the sample median), rural versus urban residence, physical and mental health status, and religiosity. Given that HIV incidence is particularly high among South African populations living in urban informal areas [8], we further divided urban residence into formal and informal categories. We also analyzed HCT across the nine provinces, which differ markedly in HIV prevalence (ranging from 7.8% in the Western Cape to 27.9% in KwaZulu-Natal among individuals aged 15 to 49 years olds) [8]. Sample weights were used to achieve nationally representative estimates.

To assess determinants of first-time testing during the campaign, we estimated multivariable logistic models regressing a binary indicator of first-time testing on demographic, socio-economic, health and geographic covariates. The sample of interest for these models was those black African individuals surveyed in both waves who had reported never having been tested for HIV in the 2010/2011 wave. These models were estimated separately by gender and separately for those residing in urban informal areas. Standard errors were estimated to account for the survey design.

To assess whether sample attrition may have biased our regression estimates of the determinants of first-time testing, we (1) examined differences in the baseline characteristics for the cross section and panel sample and (2) assessed whether the odds of being lost to follow-up in the survey varied by 2010/2011 HIV testing history.

## Results

### Characteristics of the sample

Table 1 displays descriptive statistics for the 2010/2011 cross-sectional sample. The black African population made up 79.5% of the sample, and just over half (53.9%) of the sample was comprised of women. The average respondent was 36.6 years old and had completed 9.1 years of schooling; 41.2% reported per capita household income below the national poverty line.

**Table 1:** 2010/2011 sample characteristics

Gender	Male	46.1%	[44.8%, 47.4%]
	Female	53.9%	[52.6%, 55.2%]
Race	Black African	79.5%	[74.1%, 84.9%]
	Coloured	8.5%	[4.7%, 12.4%]
	Asian/Indian	2.3%	[0.1%, 4.5%]
	White	9.7%	[6.3%, 13.1%]
Age	Mean	36.6	[35.9, 37.3]
Per capita household income (Rand)	Mean	3301^a^	[1561, 5040]
Per capita household expenditure (Rand)	Mean	2022	[1608, 2437]
Poverty	% per capita HH income <R661^b^	41%	[0.38, 0.45]
Education	Mean	9.1	[8.8, 9.3]
Currently enrolled in education	% Enrolled	15.1%	[13.8%, 16.3%]
Employment status	Employed	37.9%	[35.6%, 40.3%]
	Unemployed (broad)	14.1%	[12.4%, 15.7%]
	Not economically active	48%	[45.6%, 50.4%]
Subjective health	% “fair”/“poor”	9.7%	[8.6%, 10.7%]
Mental health	Mean CES-D 8 Score	3.78	[3.52, 4.05]
Relationship status	% married/cohabiting	36.6%	[34%, 39.2%]
Alcohol usage	% at least “drink very rarely”	26.4%	[24.2%, 28.6%]
Religious importance	% “significant”/“very significant”	90.3%	[88.7%, 91.8%]
Geographical location	Rural	39.8%	[33.6%, 46%]
	Urban formal	50.1%	[43.7%, 56.5%]
	Urban informal	10.1%	[4.9%, 15.3%]
Province	Western Cape	9.7%	[5.3%, 14.2%]
	Eastern Cape	11.9%	[7.9%, 15.9%]
	Northern Cape	2.3%	[1.4%, 3.2%]
	Free State	5.7%	[3.4%, 8%]
	KwaZulu-Natal	19.7%	[14.3%, 25.1%]
	North West	6.8%	[4.2%, 9.5%]
	Gauteng	25.4%	[18.5%, 32.3%]
	Mpumalanga	8%	[5.1%, 11%]
	Limpopo	10.3%	[6.8%, 13.9%]
Number of observations			16,683

Notes: 95% confidence intervals in brackets.

a The US dollar equivalent (as of 30 June 2010) was $253

b the US dollar equivalent (as of 30 June 2010) was $50.70. Variable descriptions are provided in the main text and Supplementary Table 1. CES-D 8, Center for Epidemiologic Studies eight-item depression scale.

### Large increases in HIV testing during the period of the national HCT campaign

**Figure 1:**
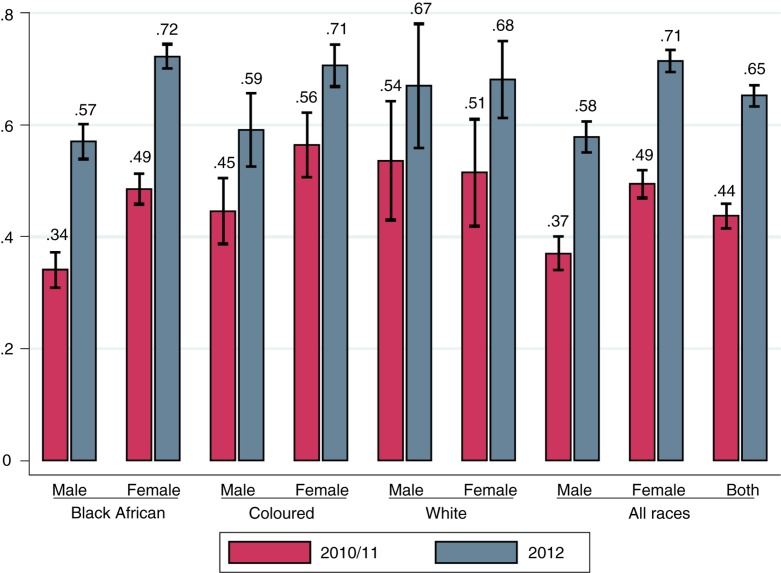
HIV testing among the full sample of individuals (15 years and older) and by gender and race. Cross-sectional data from 2010/2011 and from 2012 weighted using the NIDS cross-sectional weights; 95% confidence intervals are displayed.

Figure 1 displays population-weighted estimates of the proportion ever tested for HIV by survey wave for the full sample and separately by race and gender. For the full sample, we found that the proportion tested for HIV increased from 43.7% (95% confidence interval (CI): 41.48, 45.96) in 2010/2011 to 65.2% (95% CI: 63.28, 67.10) in 2012. Expanding by cross-sectional weights, we estimate that 13,040,000 (95% CI: 11,540,000; 14,540,000) individuals in South Africa had tested by 2010/2011 and 20,630,000 (95% CI: 18,400,000; 22,850,000) by 2012. These estimates imply that approximately 7.6 million individuals (95% CI: 6,387,910; 8,782,986) were tested for the first time over the survey period.

**Figure 2:**
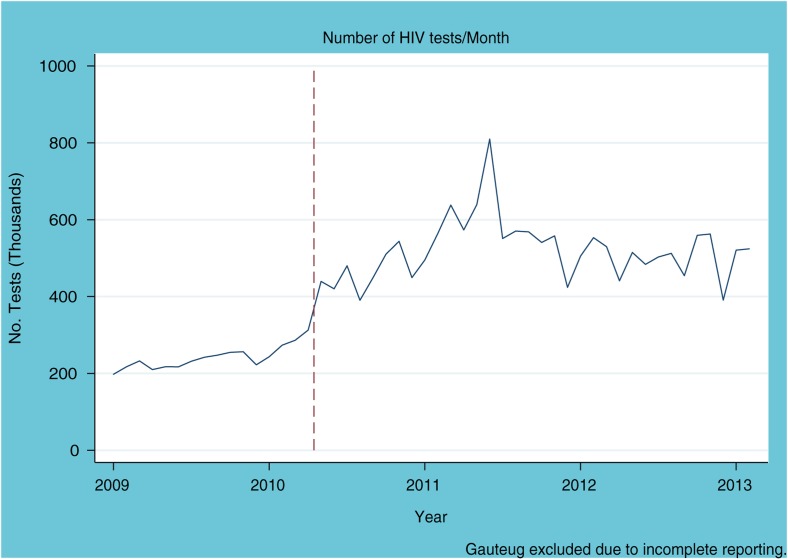
Number of HIV tests conducted per month in South Africa (aggregate data excluding data from Gauteng Province) between January 2009 and January 2013. The vertical red line indicates the launch of the HIV counselling and testing campaign.

Analysis of Department of Health data on the number of tests administered per month reveals a sharp increase starting at the time of the campaign, with a flat trend in the 14 months prior to the start of the testing campaign (Figure 2). This suggests that pre-existing trends may not appreciably account for the changes in testing rates noted in this aggregate, national analysis.

Figure 1 also demonstrates substantial increases in HIV testing coverage across gender and race categories. In 2010/2011, despite bearing the majority of the HIV burden, the proportion of black Africans who had ever tested for HIV was the lowest among all racial groups. By 2012, a large number of African women tested for the first time, and racial testing gradients for women disappeared. However, racial disparities in testing remained for men, with more than 40% of black African men remaining untested.

**Figure 3:**
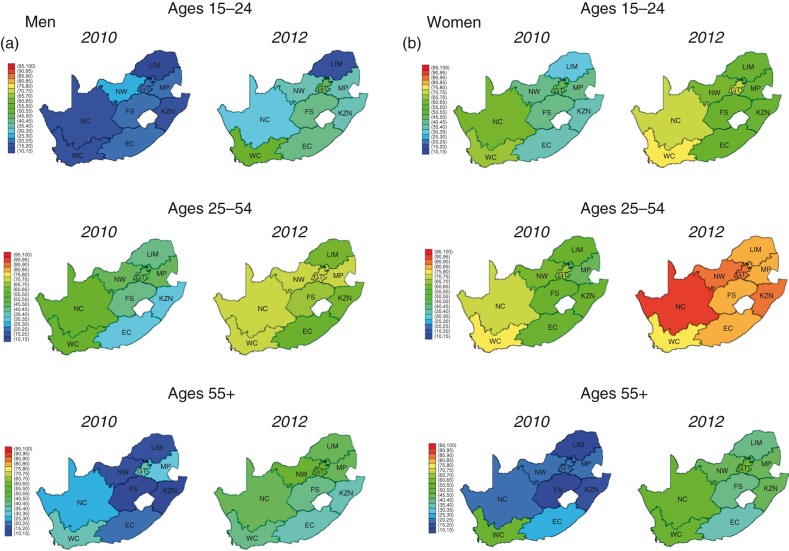
Proportions of (a) Black African men and (b) Black African women ever tested by province of residence and age group. WC, Western Cape; NC, Northern Cape; EC, Eastern Cape; FS, Free State; KZN, KwaZulu-Natal; NW, North West; GT, Gauteng; MP, Mpumalanga; LIM, Limpopo.

### HIV testing among black African individuals

Table 2 presents HIV testing estimates for black African men and women in both 2010/2011 and 2012 for different socio-economic and demographic groups. In 2010/2011, across all sub-groups, men were consistently less likely to test than women. Proportions ever tested for HIV prior to the study period were also lower among younger, less educated, poorer and less religious sub-groups. In addition, men in good health were also less likely to have tested for HIV. Multivariable logistic regression analyses (see Supplementary Table 2) showed that these pre-campaign bivariate associations with HIV testing remained robust in magnitude in multivariable models. While the bivariate association between HIV testing and health in 2010/2011 indicates that women in poorer health were less likely to have been tested for HIV, the multivariable regression analysis found the opposite result: women who reported being in poor health had significantly *greater* odds of ever testing (adjusted odds ratio (aOR): 1.69, *p*<0.001).

**Table 2:** HIV testing rates for black African men and women in 2010/2011 and 2012

	Men				Women			
		
	2010/2011			2012		2010/2011		2012	
All	34.1%	[31%, 37.3%]	57%	[53.9%, 60.1%]	48.5%	[45.8%, 51.2%]	72.2%	[70%, 74.4%]
Age group								
15 to 24	20.4%	[17.8%, 23.1%]	38.7%	[35.1%, 42.3%]	42%	[38.6%, 45.4%]	60.1%	[56%, 64.2%]
25 to 54	44%	[39.7%, 48.2%]	68.5%	[65.1%, 71.9%]	59.6%	[56.5%, 62.7%]	84.7%	[82.8%, 86.6%]
55+	22.9%	[17.1%, 28.8%]	46.1%	[40.7%, 51.5%]	20.2%	[15.9%, 24.5%]	47.8%	[43.9%, 51.7%]
Education								
Less than Grade 9	25.8%	[21.1%, 30.6%]	44%	[40%, 47.9%]	32.8%	[29.9%, 35.7%]	57.7%	[54.9%, 60.5%]
Grades 9 to 11	33.4%	[29.5%, 37.4%]	55.6%	[51.4%, 59.8%]	53.6%	[49.8%, 57.4%]	75.5%	[72.9%, 78.1%]
Grade 12 (matric)	41.6%	[35.8%, 47.5%]	69.9%	[64.9%, 74.9%]	63%	[58.3%, 67.8%]	86.2%	[83.5%, 88.9%]
Tertiary	57%	[47.7%, 66.4%]	81.7%	[76.2%, 87.1%]	66.1%	[57.9%, 74.3%]	83.4%	[74.6%, 92.2%]
Bachelor's degree	52.2%	[31.6%, 72.9%]	79.8%	[67.8%, 91.9%]	76.4%	[63.9%, 88.8%]	94%	[89.9%, 98.2%]
Poverty								
^a^Per capita household income <R661	23%	[19.9%, 26%]	45%	[41.4%, 48.5%]	43%	[40.1%, 46%]	70.6%	[68.2%, 73%]
Per capita household income >R661	42.1%	[38%, 46.2%]	62.6%	[59.4%, 65.8%]	55.3%	[52%, 58.7%]	73.5%	[70.5%, 76.4%]
Self-reported health								
Poor	40.8%	[34.6%, 47%]	53.7%	[46.6%, 60.8%]	45.4%	[39.9%, 50.9%]	65.8%	[62%, 69.7%]
Good	33.7%	[30.4%, 36.9%]	57.3%	[54.1%, 60.5%]	49%	[46.1%, 51.8%]	73.1%	[70.8%, 75.5%]
Religion								
Unimportant	25.9%	[20.1%, 31.7%]	53.9%	[46%, 61.8%]	41.8%	[30.8%, 52.8%]	68.4%	[62.2%, 74.6%]
Important	35.4%	[32%, 38.8%]	57.4%	[54.5%, 60.3%]	49%	[46.4%, 51.5%]	72.4%	[70.1%, 74.7%]
Geographical location								
Rural	27.1%	[23.3%, 31%]	47.4%	[43.8%, 51.1%]	41.3%	[37.9%, 44.6%]	67.3%	[64.6%, 69.9%]
Urban informal	27.4%	[20.3%, 34.4%]	63%	[54.2%, 71.8%]	53.4%	[47%, 59.8%]	77.6%	[71.5%, 83.7%]
Urban formal	43.7%	[38.4%, 49%]	64.2%	[60.2%, 68.2%]	56%	[52.1%, 59.9%]	76.3%	[73.4%, 79.2%]
Number of observations	5762		6189		8182		9164	

Notes: 95% confidence intervals in square brackets. Each cell represents the proportion of individuals in the denoted sample and survey year reporting ever having been tested for HIV. ^a^The US dollar equivalent (as of 30 June 2010) was $50.70. Variable descriptions are provided in the main text and Supplementary Table 1.

During the study period, relatively large increases (≥15% points) in HIV testing were observed in all male and female sub-groups. Particularly large improvements were observed among men living in urban informal areas, women older than 54 and women below the poverty line. Smaller improvements were seen for young (15 to 24) and less educated (less than Grade 9) men: among young and less educated men who had not been tested in 2010/2011, only 23 and 25%, respectively, tested during the next two years.

In 2012, proportions ever tested for HIV were relatively low among several groups. Among black African men, proportions ever tested for HIV were low for younger (15 to 24), less educated and poorer individuals and for those living in rural areas. Among black African women, the younger (15 to 24), older (55+) and least-educated individuals reported less HIV testing.

We found considerable geographic heterogeneity in testing in 2010/2011 for all groups that, despite significant reductions in differences between provinces during the study period, remained evident in 2012 (Figure 3). Among young men, for example, the lowest and highest proportions who had ever tested in 2012 were 19.2% (95% CI: 19.1%, 19.4%) in Limpopo Province and 56.7% (95% CI: 56.4%, 57%) in the Western Cape. Notably, testing rates were not necessarily the highest in the highest HIV prevalence regions (2012 HIV prevalence >19% among individuals aged 15 to 49): KwaZulu-Natal, Mpumalanga, Free State, North West and the Eastern Cape [8]. In addition, testing remained relatively low among older populations, with few provinces having tested more than 50% of this population by 2012. (District-level analysis highlighted large within-province variation in testing coverage – see Supplementary Figure 3).

### Predictors of first-time testing during the campaign

Table 3 presents the output from our multivariable analysis of uptake of HIV testing among black African men and women between 2010 and 2012. Model 1, for the full black African sample, shows that women had significantly greater odds than men of testing for the first time between 2010 and 2012 (aOR: 1.9; *p*<0.001). Models 2 and 3 show that, for both men and women, older and more educated individuals had significantly greater odds of testing for the first time. For example, complementary analysis using education categories showed that men who had completed high school had 1.87 (*p*<0.001) greater odds of testing for the first time compared to men with lower than Grade 9 education (available upon request). These results suggest that the large disparities in HIV testing by gender, age and education that existed prior to the campaign (see Supplementary Table 2) widened further during the campaign.

**Table 3:** Logistic regression models of factors associated with first-time HIV testing among black African men and women between 2010 and 2012

	Full sample	Men	Women
	1	2	3
Model	aOR [95% CI]	aOR [95% CI]	aOR [95% CI]
Female	1.927***	na	na
	[1.594 to 2.330]	na	na
Age	1.075***	1.073***	1.054***
	[1.041 to 1.110]	[1.027 to 1.121]	[1.014 to 1.095]
Age squared	0.999***	0.999***	0.999***
	[0.999 to 0.999]	[0.999 to 1.000]	[0.999 to 1.000]
Log real per capita household income	1.006	1.086	0.91
	[0.917 to 1.104]	[0.950 to 1.243]	[0.807 to 1.028]
Years of education	1.115***	1.138***	1.099***
	[1.086 to 1.144]	[1.085 to 1.195]	[1.062 to 1.136]
Currently enrolled in school	0.755*	0.908	0.498***
	[0.566 to 1.005]	[0.612 to 1.347]	[0.333 to 0.744]
Unemployed (base=employed)	1.054	0.967	1.158
	[0.757 to 1.468]	[0.628 to 1.491]	[0.771 to 1.738]
Economically inactive (base=employed)	1.141	1.221	1.068
	[0.848 to 1.535]	[0.844 to 1.766]	[0.780 to 1.462]
Married/cohabitating	1.250*	1.144	1.213
	[0.989 to 1.581]	[0.810 to 1.615]	[0.891 to 1.653]
Religion very important	1.064	1.034	1.147
	[0.919 to 1.231]	[0.859 to 1.244]	[0.966 to 1.362]
Poor/fair health (base=good/excellent)	0.803	0.891	0.802
	[0.611 to 1.056]	[0.554 to 1.431]	[0.572 to 1.123]
CES-D 8 scale	0.992	0.986	1
	[0.968 to 1.016]	[0.954 to 1.019]	[0.973 to 1.028]
Drinks alcohol	1.217*	1.207	1.182
	[0.975 to 1.520]	[0.899 to 1.621]	[0.754 to 1.853]
Rural (base=urban formal)	1.077	1.042	1.151
	[0.810 to 1.433]	[0.718 to 1.513]	[0.786 to 1.685]
Urban informal (base=urban formal)	1.322	1.26	1.514
	[0.788 to 2.217]	[0.784 to 2.027]	[0.680 to 3.375]
Days between interview	0.999	0.998*	1
	[0.997 to 1.000]	[0.996 to 1.000]	[0.998 to 1.001]
Pregnant between waves	2.536***	na	2.518***
	[1.978 to 3.252]	na	[1.963 to 3.230]
Controls for province of residence	Yes	Yes	Yes
Observations	6081	2708	3373

Notes:

*** *p*<0.01

* *p*<0.1; 95% confidence intervals in brackets. Each column represents a separate logistic regression. The sample of interest is described in the header. All samples are restricted to those individuals surveyed in both 2010/2011 and 2012 who reported never having been tested for HIV in the former survey wave. As such, these regressions assess the determinants of first-time testing by 2012 among the sample of never-testers in 2010/2011. CES-D 8, Center for Epidemiologic Studies eight-item depression scale; CI, confidence interval; aOR, adjusted odds ratio.

On the other hand, no statistically significant relationship was found among men and women between first-time HIV testing during the campaign and either *per capita* household income, religiosity or self-reported health. This finding stands in contrast to patterns prior to the campaign (see Supplementary Table 2), and suggests an improvement in access to HCT with uptake of HCT during the campaign period no longer being determined by wealth, religiosity or poor health.

We also examined determinants of first-time testing among black African residents in urban informal areas due to particularly high HIV incidence rates in these areas [8]. Consistent with results among all black African individuals, women, more educated individuals and more religious individuals were more likely to have tested for the first time during the study period (Supplementary Table 4). Notably, urban informal residents who reported greater levels of depression were less likely to have been a first-time tester. In addition, we assessed factors associated with first-time testing among pregnant and non-pregnant black African women due to large differentials in testing between these groups (Supplementary Table 4). Focusing on previously untested women, 74% who were pregnant during the study period tested for the first time compared to 53% who did not report a pregnancy. We found no substantive or statistically significant predictors of first-time testing among pregnant women; however, among non-pregnant women, we found similar age and education gradients as in Table 3.

Finally, our sensitivity analyses showed that, with the exception of gender (a greater proportion of men left the study), our cohort sample was not substantively influenced by attrition on key demographic and socio-economic measures. No evidence of attrition bias was found in the dependent (HIV testing) variable (both sets of results available upon request).

## Discussion

In this study, we used new nationally representative data from South Africa to assess the impact of a 15-month national HIV testing campaign. We specifically focused on two major aims of the campaign: uptake of HIV testing by persons who had not previously tested and uptake of testing by populations at high risk for HIV [10]. The numbers of first-time testers reached by the campaign and its level of success in targeting high-risk populations have not been previously measured. We estimate that approximately 7.6 million South Africans self-reported testing for the first time during the intervention. Given that an estimated 20 million HIV tests were conducted between April 2010 and December 2011, our estimates suggest that approximately one in three tests conducted during the campaign were for someone who had not previously tested for HIV. This is a remarkable achievement given early warnings that testing uptake had been underwhelming [33].

Overall, HIV testing increased among higher-risk populations, suggesting that the campaign may have improved targeting of HIV testing. Focusing on black Africans, our findings indicate large increases in self-reported testing rates among poorer individuals, who are also at greater risk for HIV infection [8], as well as individuals who may be more socially isolated (those not belonging to community groups such as religious organizations). Improved access among these populations may have been due to the expansion of HIV testing services into communities through activities such as home-based testing, which has been shown to be particularly effective in reaching poorer individuals [34] and first-time testers [35], and the use of mobile clinics, which are also effective in reaching populations not previously tested for HIV [36]. Moreover, the average first-time tester during the campaign was healthier (based on self-reported measures), suggesting that testing increased among people who were not already very sick, a *sine qua non* for the prevention benefits of treatment.

However, the findings suggest key shortfalls. First, by 2012, 35% of the black African population aged 15 and older had never been tested for HIV, a finding that is consistent with data from other national surveys [8,37]. Much of this shortfall appears to have been driven by unmitigated and potentially widening disparities in testing by gender and level of education, as well as low rates of testing among those aged 15 to 24. Our finding that gender disparity in HIV testing could be increasing is consistent with other research indicating that HCT uptake has increased more substantially in women than men [38].

There are several limitations to our study. First, our estimate of first-time testers is based on self-reported HIV testing in two time periods. Although our 2012 estimates of testing coverage are consistent with other surveys (e.g. the South African National HIV Prevalence, Incidence and Behaviour Survey) conducted in 2012 [8], it is still possible that our estimates reflect social desirability bias [38]. Moreover, estimates of first-time testers would also be biased if the accuracy of self-reports of HIV testing changed over time. This outcome would be plausible during a national HCT campaign aiming to normalize HCT if individuals who might have been reluctant to report HIV testing prior to the campaign (due to factors such as stigma) subsequently had a tendency to over-report testing behaviour. However, evidence from mathematical models indicates that self-reported HCT data collected in surveys both prior to and after the national HCT campaign were overestimates of HIV testing coverage [38], suggesting that a change of this nature did not occur over the campaign period.

Second, as discussed above, the first wave of NIDS data was collected *during* the national testing campaign. As such, our results may underestimate the number of new testers. A cross-cutting source of bias is the fact that we cannot be sure if the differences we find across waves were due to the campaign itself or pre-existing trends in testing. Although we demonstrated a flat trend in the number of tests per month nationally in the 14 months prior to the campaign, it is possible that there were pre-existing trends in the number of new testers within key sub-groups. Third, given the lack of data we were unable to evaluate several potentially important determinants of HIV testing – such as HIV knowledge, perceived stigma and attitudes to and knowledge of HIV testing – and potential mechanisms linking socio-economic characteristics to testing uptake, all of which may be modified by testing campaign activities [39–41]. Fourth, our study does not capture potential longer-run intervention effects. Lastly, because of data limitations, our findings do not identify effects of specific programme strategies on testing rates.

Nevertheless, our results have several implications for HIV policies that aim to improve the equity of HCT uptake and thereby increase the potential efficacy of HIV prevention and treatment initiatives. First, a national scale-up of HIV testing incorporating best practices in HCT service delivery has the potential to reach priority and underserved groups and improve equity in access to HIV testing. The South African example demonstrates the power of utilizing a number of evidence-based strategies, such as the use of mobile clinics [42,43], provider-initiated HCT [44] and home-based service provision [34]. Although data limitations prevent us from assessing the relative effectiveness of these different strategies, the extensive individual and spatial heterogeneity we find in programme impacts can serve as a guide for future studies seeking to identify best practices.

Second, extensive use of evidence-based techniques may still leave out specific high-risk populations. The lower efficacy of the South African campaign in reaching less-educated individuals, particularly men, is at first glance striking given the programme's success in reaching poor individuals and urban informal residents. However, this finding is consistent with previous studies identifying more sluggish uptake of health interventions among less-educated populations [27,45–47]. Because less educated individuals are at higher risk of contracting HIV both in South Africa and elsewhere in sub-Saharan Africa [26,27,48–50], it is imperative for HCT services to reach this population. The failure of the campaign to proportionally increase testing among the less educated may, in part, have been influenced by the mass media advertising employed during the campaign, to which the educated likely had greater exposure [51]. Regardless, the findings underscore the need for the development of novel techniques to reach this population. For example, opt-out testing strategies demonstrated HCT of greater than 90% among less-educated men in the opt-out model, as opposed to 60% in an opt-in model [52]. Home-based HCT [34] and conditional economic incentives [53–55] may serve as useful adjuncts, as well.

Third, our results highlight the importance of high-resolution micro-data in both evaluating and targeting HCT campaigns. The rich spatial, demographic and socio-economic heterogeneity in campaign effects noted in this study reveal the need for developing reliable population data collection systems as part of HCT efforts. These data collection systems must also enable patient tracking, which is essential to evaluate whether the potential individual and public health benefits of HIV diagnosis and linkage to care are being realized. Without robust data, *en face* successes of ambitious programs may obscure important shortfalls.

## Conclusions

The proportion of South Africans who had ever tested for HIV increased dramatically during an extensive and unprecedented national campaign conducted between 2010 and 2011. The campaign also appeared to have improved the targeting of HCT services and equity in uptake of testing. However, increases in testing rates among men and the less educated were much less impressive. Novel interventions may be required to achieve universal HCT access and uptake in these populations.

## Supplementary Material

Changes in self-reported HIV testing during South Africa's 2010/2011 national testing campaign: gains and shortfalls

Changes in self-reported HIV testing during South Africa's 2010/2011 national testing campaign: gains and shortfalls

Changes in self-reported HIV testing during South Africa's 2010/2011 national testing campaign: gains and shortfalls

Changes in self-reported HIV testing during South Africa's 2010/2011 national testing campaign: gains and shortfalls

## References

[1] Bor J, Herbst AJ, Newell M-L, Bärnighausen T (2013). Increases in adult life expectancy in rural South Africa: valuing the scale-up of HIV treatment. Science.

[2] Johnson LF, Mossong J, Dorrington RE, Schomaker M, Hoffmann CJ, Keiser O (2013). Life expectancies of South African adults starting antiretroviral treatment: collaborative analysis of cohort studies. PLoS Med.

[3] Cohen MS, Chen YQ, McCauley M, Gamble T, Hosseinipour MC, Kumarasamy N (2011). Prevention of HIV-1 infection with early antiretroviral therapy. N Engl J Med.

[4] Bor J, Barnighausen T (2015). When to start HIV treatment: evidence from a regression discontinuity study in South Africa.

[5] UNAIDS (2014). 90-90-90: an ambitious treatment target to help end the AIDS Epidemic.

[6] Montaner JSG, Lima VD, Barrios R, Yip B, Wood E, Kerr T (2010). Association of highly active antiretroviral therapy coverage, population viral load, and yearly new HIV diagnoses in British Columbia, Canada: a population-based study. Lancet.

[7] Tanser F, Bärnighausen T, Grapsa E, Zaidi J, Newell ML (2013). High coverage of ART associated with decline in risk of HIV acquisition in Rural KwaZulu-Natal, South Africa. Science.

[8] Shisana O, Rehle T, Simbayi L (2014). South African National HIV prevalence, incidence and behaviour survey, 2012.

[9] De Cock KM, Bunnell R, Mermin J (2006). Unfinished business – Expanding HIV testing in developing countries. N Engl J Med.

[10] South African National AIDS Council (2010). The national HIV counselling and testing Campaign Strategy.

[11] Department of Health (2010). HIV counselling and testing (HCT) policy guidelines.

[12] UNAIDS (2013). Getting to zero: HIV in Eastern & Southern Africa.

[13] South African National AIDS Council (2013). Republic of South Africa Global AIDS Response Progress Report: Mid-term Review of Progress in Achieving the 2011 UN General Assembly Political Declaration on HIV/AIDS targets and elimination commitments in South Africa.

[14] Mbengashe T, Nevhutalu Z, Chipimo M, Chidarikire T, Diseko L (2012). The national HIV counselling and testing campaign and treatment expansion in South Africa: a return on investments in combination prevention.

[15] Venkatesh K, Madiba P, de Bruyn G, Lurie MN, Coates TJ, Gray GE (2011). Who gets tested for HIV in a South African Urban Township? Implications for test and treat and gender-based prevention interventions. J Acquir Immune Defic Syndr.

[16] Shisana O, Rehle T, Simbayi L, Zuma K, Jooste S, Pillay-Van Wyk V (2009). South African national prevalence, incidence, behaviour and communication survey, 2008: a turning tide among teenagers?.

[17] Johnson S, Kincaid L, Laurence S, Chikwava F, Delate R, Mahlasela L (2010). The second national HIV communication survey, 2009.

[18] Pettifor A, MacPhail C, Suchindran S, Delany-Moretlwe S (2008). Factors associated with HIV testing among public sector clinic attendees in Johannesburg, South Africa. AIDS Behav.

[19] Peltzer K, Matseke G, Mzolo T, Majaja M (2009). Determinants of knowledge of HIV status in South Africa: results from a population-based HIV survey. BMC Public Health.

[20] Ropelewski LR, Hulbert A, Latimer WW (2011). Factors related to past HIV testing among South African non-injection drug users. AIDS Care.

[21] Mhlongo S, Dietrich J, Otwombe KN, Robertson G, Coates TJ, Gray G (2013). Factors associated with not testing for HIV and consistent condom use among men in Soweto, South Africa. PLoS One.

[22] Obermeyer CM, Neuman M, Hardon A, Desclaux A, Wanyenze R, Ky-Zerbo O (2013). Socio-economic determinants of HIV testing and counselling: a comparative study in four African countries. Trop Med Int Health.

[23] Hensen B, Lewis JJ, Schaap A, Tembo M, Mutale W, Weiss HA (2015). Factors associated with HIV-testing and acceptance of an offer of home-based testing by men in rural Zambia. AIDS Behav.

[24] Creel AH, Rimal RN (2011). Factors related to HIV-testing behavior and interest in testing in Namibia. AIDS Care.

[25] Agha S (2012). Factors associated with HIV testing and condom use in Mozambique: implications for programs. Reprod Health.

[26] Bärnighausen T, Hosegood V, Timaeus I, Newell M (2007). The socioeconomic determinants of HIV incidence: evidence from a longitudinal, population-based study in rural South Africa. AIDS.

[27] De Neve J-W, Fink G, Subramanian SV, Moyo S, Bor J (2015). Length of secondary schooling and risk of HIV infection in Botswana: evidence from a natural experiment. Lancet Global Health.

[28] de Villiers L, Brown M, Woolard I, Daniels R, Leibbrandt M (2014). National income dynamics study wave 3 user manual: Southern Africa labour and development research unit.

[29] Girardi E, Aloisi MS, Arici C, Pezzotti P, Serraino D, Balzano R (2004). Delayed presentation and late testing for HIV: demographic and behavioral risk factors in a multicenter study in Italy. J Acquir Immune Defic Syndr.

[30] Crown WH, Finkelstein S, Berndt ER, Ling D, Poret AW, Rush AJ (2002). The impact of treatment-resistant depression on health care utilization and costs. J Clin Psychiatry.

[31] Wu M, LaGasse LL, Wouldes TA, Arria AM, Wilcox T, Derauf C (2012). Predictors of inadequate prenatal care in methamphetamine-using Mothers in New Zealand and the United States. Matern Child Health J.

[32] Radloff LS (1977). The CES-D scale: a self-report depression scale for research in the general population. Appl Psychol Meas.

[33] IRIN SOUTH AFRICA: National HIV testing campaign disappoints.

[34] Helleringer S, Kohler H-P, Frimpong JA, Mkandawire J (2009). Increasing uptake of HIV testing and counseling among the poorest in Sub-Saharan Countries Through home-based service provision. J Acquir Immune Defic Syndr.

[35] Doherty T, Tabana H, Jackson D, Naik R, Zembe W, Lombard C (2013). Effect of home based HIV counselling and testing intervention in rural South Africa: cluster randomised trial. BMJ Open.

[36] Mabuto T, Latka MH, Kuwane B, Churchyard GJ, Charalambous S, Hoffmann CJ (2014). Four models of HIV counseling and testing: utilization and test results in South Africa. PLoS One.

[37] Johnson S, Kincaid DL, Figueroa M, Delate R, Mahlasela L, Magni S (2013). The third national HIV communication survey, 2012.

[38] Johnson LF, Rehle TM, Jooste S, Bekker L-G (2015). Rates of HIV testing and diagnosis in South Africa: successes and challenges. AIDS.

[39] Peltzer K, Matseke G (2014). Determinants of HIV testing among young people aged 18–24 years in South Africa. Afr Health Sci.

[40] Maughan-Brown B, Nyblade L (2014). Different dimensions of HIV-related stigma may have opposite effects on HIV testing: evidence among young men and women in South Africa. AIDS Behav.

[41] Kalichman S, Simbayi L (2003). HIV testing attitudes, AIDS stigma, and voluntary HIV counselling and testing in a black township in Cape Town, South Africa. Sex Transm Infect.

[42] van Schaik N, Kranzer K, Wood R, Bekker L-G (2010). Earlier HIV diagnosis – are mobile services the answer?. S Afr Med J.

[43] Maheswaran H, Thulare H, Stanistreet D, Tanser F, Newell M-L (2012). Starting a home and mobile HIV testing service in a rural area of South Africa. J Acquir Immune Defic Syndr.

[44] Kennedy CE, Fonner VA, Sweat MD, Okero FA, Baggaley R, O'Reilly KR (2013). Provider-initiated HIV testing and counseling in low- and middle-income countries: a systematic review. AIDS Behav.

[45] De Walque D (2009). Does education affect HIV status? Evidence from five African Countries. World Bank Econ Rev.

[46] Cutler DM, Lleras-Muney A (2010). Understanding differences in health behaviors by education. J Health Econ.

[47] Gummerson E, Maughan-Brown B, Venkataramani A (2013). Who is taking up voluntary medical male circumcision? Early evidence from Tanzania. AIDS.

[48] Gummerson E (2013). Have the educated changed HIV risk behaviours more in Africa?. AJAR.

[49] Behrman JA (2015). The effect of increased primary schooling on adult women's HIV status in Malawi and Uganda: universal primary education as a natural experiment. Soc Sci Med.

[50] Hargreaves JR, Bonell CP, Boler T, Boccia D, Birdthistle I, Fletcher A (2008). Systematic review exploring time trends in the association between educational attainment and risk of HIV infection in sub-Saharan Africa. AIDS.

[51] Peltzer K, Parker W, Mabaso M, Makonko E, Zuma K, Ramlagan S (2012). Impact of national HIV and AIDS communication campaigns in South Africa to Reduce HIV risk behaviour. Sci World J.

[52] Baisley K, Doyle AM, Changalucha J, Maganja K, Watson-Jones D, Hayes R (2012). Uptake of voluntary counselling and testing among young people participating in an HIV prevention trial: comparison of opt-out and opt-in strategies. PLoS One.

[53] Thornton RL (2008). The demand for, and impact of, learning HIV status. Am Econ Rev.

[54] Galárraga O, Genberg BL, Martin RA, Barton Laws M, Wilson IB (2013). Conditional economic incentives to improve HIV treatment adherence: literature review and theoretical considerations. AIDS Behav.

[55] Baird SJ, Garfein RS, McIntosh CT, Ozler B (2012). Effect of a cash transfer programme for schooling on prevalence of HIV and herpes simplex type 2 in Malawi: a cluster randomised trial. Lancet.

