# Supplementary material for: Changes in self-reported HIV testing during South Africa's 2010/2011 national testing campaign: gains and shortfalls
Source: J Int AIDS Soc. 2016 Apr 11;19(1):20658. doi: 10.7448/IAS.19.1.20658 (PMC4829657; doi:10.7448/IAS.19.1.20658)
Supplement: Changes in self-reported HIV testing during South Africa's 2010/2011 national testing campaign: gains and shortfalls [file JIAS-19-20658-s001.pdf]

Additional File 1

Table A1: Description of the Independent variables used in the analysis

**Demographic and Socioeconomic Variables**	
Gender	Males are the base category.
Age	Age in years as both a linear and quadratic variable
Race	The four main population groups in South Africa are included: African (referring to black Africans), Coloured, Indian/Asian, and White. 'Coloured' is a common and socially acceptable term in South Africa for individuals of mixed race. Africans are the base category.
Labour market status	A broad definition of unemployment is used (combining the searching and non-searching unemployed). Three categories are defined: employed, unemployed and not-economically-active, with the employed as the base category.
Income	Income is used in log form and is calculated using NIDS's household income variable (with full imputations) divided by household size. See Argent (2009) for a full discussion on the construction of this variable.
Poverty	An individual is defined as poor if their per capita real household income is below R662 (in 2010 rands). This poverty line was based on 2008 poverty line of R515 (approximately $62 in 2008) which is widely used in the literature (Woolard and Leibbrandt, 2005).
Enrolment	The NIDS questionnaire asked all individuals under the age of 30 whether or not they are currently enrolled in an education institution. We created a binary variable = 1 for individuals who were enrolled at the time of the interview and make the assumption that individuals older than 30 were not enrolled.
Education	The education variable represents years of completed education. The range of the variable is 0 to 18 (someone with more than a Bachelor's degree).
Marital status	A binary variable = 1 for individuals who were married or living with their partner and 0 otherwise. Note, this variable does not identify whether or not an individual is involved in a sexual relationship with anyone, only that they are either married or living with their partner. The variable is derived directly from the NIDS household roster.
Religious Intensity	Respondents were asked the following question about religion "How important are religious activities in your life? A binary variable was created =1 for the response "important" or "very important" and 0 for "unimportant" or "not important at all".
Province	There are 9 provinces in South Africa. The base category is KwaZulu-Natal, the province with the highest HIV prevalence in South Africa (Shisana et al., 2014).
Geographical location	Geographical location of current residence was coded into four categories: rural formal, tribal authority area, urban formal and urban informal. Rural formal area refers to predominantly commercial farms, whereas tribal authority area refers to rural areas outside commercial farms with a mixture of traditional and civil authority. Urban formal area refers to areas close to commercial centres with physical infrastructure and formal urban planning, whereas urban informal area refers to informal settlements close to commercial centres with no physical infrastructure or formal urban planning. Urban informal is the base category.
**Health variables**	
Subjective health	Self-perceived health was measured using an ordinal scale from 1-5 where 1 is "excellent" and 5 "poor". Interviewees were asked the question, "How would you describe your health at present? Would you say it is excellent, very good, good, fair, or poor?" A binary variable was created to identify individuals who reported "poor" or "fair" (=1) and those who reported "excellent", "very good" or "good" (=0).
Depression scale	NIDS asked the standard 10 questions used to form the CESD scale. Previous analysis indicates that responses to two of these questions were influenced by the response option running in the opposite direction compared to the other 8 questions (Ardington and Case, 2010). We therefore dropped these two questions and use a continuous 8-question scale as a measure of mental health.<br>The ten questions are asked in the form of phrases with which the respondents are asked to rank their agreement on a scale of 1 to 4 with1 meaning "Rarely or none of the time" and 4 meaning "All of the time". The eight questions used to construct our mental health scale were:<br>(1) I was bothered by things that usually don't bother me; (2) I had trouble keeping my mind on what I was doing; (3) I felt depressed; (4) I felt that everything I did was an effort; (5) I felt fearful; (6) My sleep was restless; (7) I felt lonely; (8) I could not "get going"<br><br>The two inverted, and therefore excluded, questions were (1) I felt hopeful about the future, and (2) I was happy.
Alcohol usage	Alcohol usage was reported on an ordinal scale of 1-7 where 1 represented "I have never drank alcohol" and 8 "every day". A binary variable was created with a value of 1 representing individuals who responded at least "rarely" (scores 3-8) and 0 representing individuals who reported never having consumed alcohol or had stopped drinking.
Pregnant	A binary indicator or whether women reported giving birth between the surveys.
