# Supplementary material for: Changes in self-reported HIV testing during South Africa's 2010/2011 national testing campaign: gains and shortfalls
Source: J Int AIDS Soc. 2016 Apr 11;19(1):20658. doi: 10.7448/IAS.19.1.20658 (PMC4829657; doi:10.7448/IAS.19.1.20658)
Supplement: Changes in self-reported HIV testing during South Africa's 2010/2011 national testing campaign: gains and shortfalls [file JIAS-19-20658-s002.pdf]

## Additional File 2

**Table A2:** Logistic regression models of factors associated with HIV testing among the full African sample in 2010/11

| | **Full sample** | **African men** | **African women** |
|---|---|---|---|
| Model | 1 | 2 | 3 |
| Female | 2.262*** | | |
| | [1.963 - 2.607] | | |
| Age | 1.109*** | 1.139*** | 1.088*** |
| | [1.072 - 1.147] | [1.086 - 1.195] | [1.045 - 1.133] |
| Age squared | 0.998*** | 0.998*** | 0.999*** |
| | [0.998 - 0.999] | [0.998 - 0.999] | [0.998 - 0.999] |
| Log real pc household income | 1.211*** | 1.329*** | 1.124** |
| | [1.120 - 1.309] | [1.185 - 1.491] | [1.027 - 1.230] |
| Years of education | 1.087*** | 1.072*** | 1.098*** |
| | [1.063 - 1.112] | [1.036 - 1.110] | [1.069 - 1.128] |
| Enrolled in education | 0.467*** | 0.606*** | 0.355*** |
| | [0.377 - 0.579] | [0.414 - 0.886] | [0.270 - 0.467] |
| Unemployed (base = employed) | 0.836* | 0.707** | 0.968 |
| | [0.689 - 1.015] | [0.501 - 0.999] | [0.765 - 1.224] |
| Not economically active (base = employed) | 0.788** | 0.901 | 0.798* |
| | [0.647 - 0.960] | [0.699 - 1.162] | [0.635 - 1.004] |
| Married/cohabitating | 1.458*** | 1.588*** | 1.228** |
| | [1.248 - 1.704] | [1.246 - 2.024] | [1.006 - 1.498] |
| Religion very important | 1.499*** | 1.473** | 1.565* |
| | [1.106 - 2.032] | [1.090 - 1.991] | [0.953 - 2.569] |
| Poor/fair health (base = good/excellent) | 1.650*** | 1.741*** | 1.685*** |
| | [1.321 - 2.062] | [1.238 - 2.449] | [1.283 - 2.211] |
| CESD 8 scale | 1.006 | 1.020 | 0.998 |
| | [0.987 - 1.024] | [0.993 - 1.048] | [0.977 - 1.018] |
| Drinks alcohol | 1.081 | 1.059 | 1.136 |
| | [0.889 - 1.314] | [0.829 - 1.353] | [0.861 - 1.499] |
| Rural (base = urban formal) | 0.855 | 0.817 | 0.921 |
| | [0.672 - 1.089] | [0.589 - 1.134] | [0.721 - 1.176] |
| Urban Informal (base = urban formal) | 0.846 | 0.681* | 1.071 |
| | [0.650 - 1.100] | [0.456 - 1.016] | [0.767 - 1.495] |
| Control for Province of residence | Yes | Yes | Yes |
| Observations | 13,213 | 5,447 | 7,766 |
| Pseudo R-squared | 0.137 | 0.127 | 0.143 |

Notes: 95% Confidence Intervals in brackets
*** p<0.01, ** p<0.05, * p<0.1
A full description of all the independent variables can be found in Additional File 2.
