# Supplementary material for: Changes in self-reported HIV testing during South Africa's 2010/2011 national testing campaign: gains and shortfalls
Source: J Int AIDS Soc. 2016 Apr 11;19(1):20658. doi: 10.7448/IAS.19.1.20658 (PMC4829657; doi:10.7448/IAS.19.1.20658)
Supplement: Changes in self-reported HIV testing during South Africa's 2010/2011 national testing campaign: gains and shortfalls [file JIAS-19-20658-s003.pdf]

Additional File 3

In the figures below we display a more detailed view of the geographic variation in HIV testing coverage by providing unconditional probabilities of testing at the district level in 2012. The sample is restricted to black Africans. Figure A1 displays a map for the African adult population. Figure A2 displays maps for African men and women separately. We only report the results for districts with a sample size of at least 100 for the total adult population map and at least 50 observations for the male and female maps. The districts without sufficient observations were largely in the Western Cape, Northern Cape and parts of the Eastern Cape. The districts within provinces with a higher HIV prevalence did have sufficient data. The maps highlight the large variation in HIV testing coverage across districts. Within KwaZulu-Natal there appears to be areas where about 50% of the adult population was still untested in 2012 – for example, Umgungundlovu District (DC22) which Shisana et al. (2014) show to have a very high HIV prevalence. Similarly, most districts in Limpopo province had a coverage rate of about 50%, while districts in North West, Mpumalanga and Free State were within the 60-70% range in 2012. Testing coverage was the highest within Gauteng districts.

Figure A2 displays the district level coverage rates in 2012 by gender. The areas of high and low coverage were consistent across gender in 2012, but almost all districts had a higher female coverage rate. For men, a number of districts in the Northern parts of Limpopo province had a low coverage rate, together with a few districts in KwaZulu-Natal and northern parts of the Eastern Cape. For women, the districts with a lower coverage rate in 2012 were largely found along the Eastern side of the country, in Limpopo, Mpumalanga, KwaZulu-Natal and north-eastern parts of the Eastern Cape.

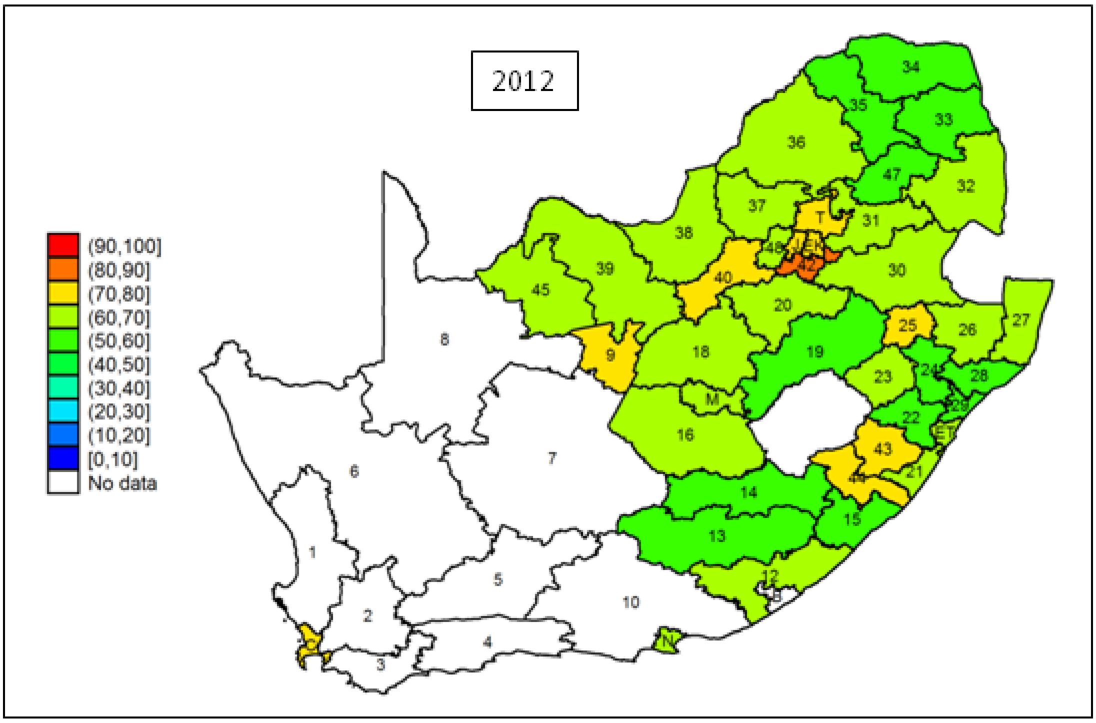

Figure A1: Cross-sectional data from 2012 showing the proportion of African individuals (15 years and older) ever tested for HIV in each district. Districts with less than 100 observations are not included. The data is weighted using the NIDS cross-sectional weights. See labels below.

District Labels

DC1	West Coast
DC2	Cape Winelands
DC3	Overberg
DC4	Eden
DC5	Central Karoo
DC6	Namakwa
DC7	Pixley ka Seme
DC8	Siyanda
DC9	Frances Baard
DC10	Cacadu
DC12	Amathole
DC13	Chris Hani
DC14	Joe Gqabi
DC15	O.R.Tambo
DC16	Xhariep
DC18	Lejweleputswa
DC19	Thabo Mofutsanyane
DC20	Fezile Dabi

DC21	Ugu
DC22	UMgungundlovu
DC23	Uthukela
DC24	Umzinyathi
DC25	Amajuba
DC26	Zululand
DC27	Umkhanyakude
DC28	Uthungulu
DC29	iLembe
DC30	Gert Sibande
DC31	Nkangala
DC32	Ehlanzeni
DC33	Mopani
DC34	Vhembe
DC35	Capricorn
DC36	Waterberg
DC37	Bojanala
DC38	Ngaka Modiri Molema

DC39	Dr Ruth Segomotsi Mompati
DC40	Dr Kenneth Kaunda
DC42	Sedibeng
DC43	Sisonke
DC44	Alfred Nzo
DC45	John Taolo Gaetsewe
DC47	Greater Sekhukhune
DC48	West Rand
BUF	Buffalo City
CPT	City of Cape Town
EKU	Ekurhuleni
ETH	eThekwini
JHB	City of Johannesburg
MAN	Mangaung
NMA	Nelson Mandela Bay
TSH	City of Tshwane

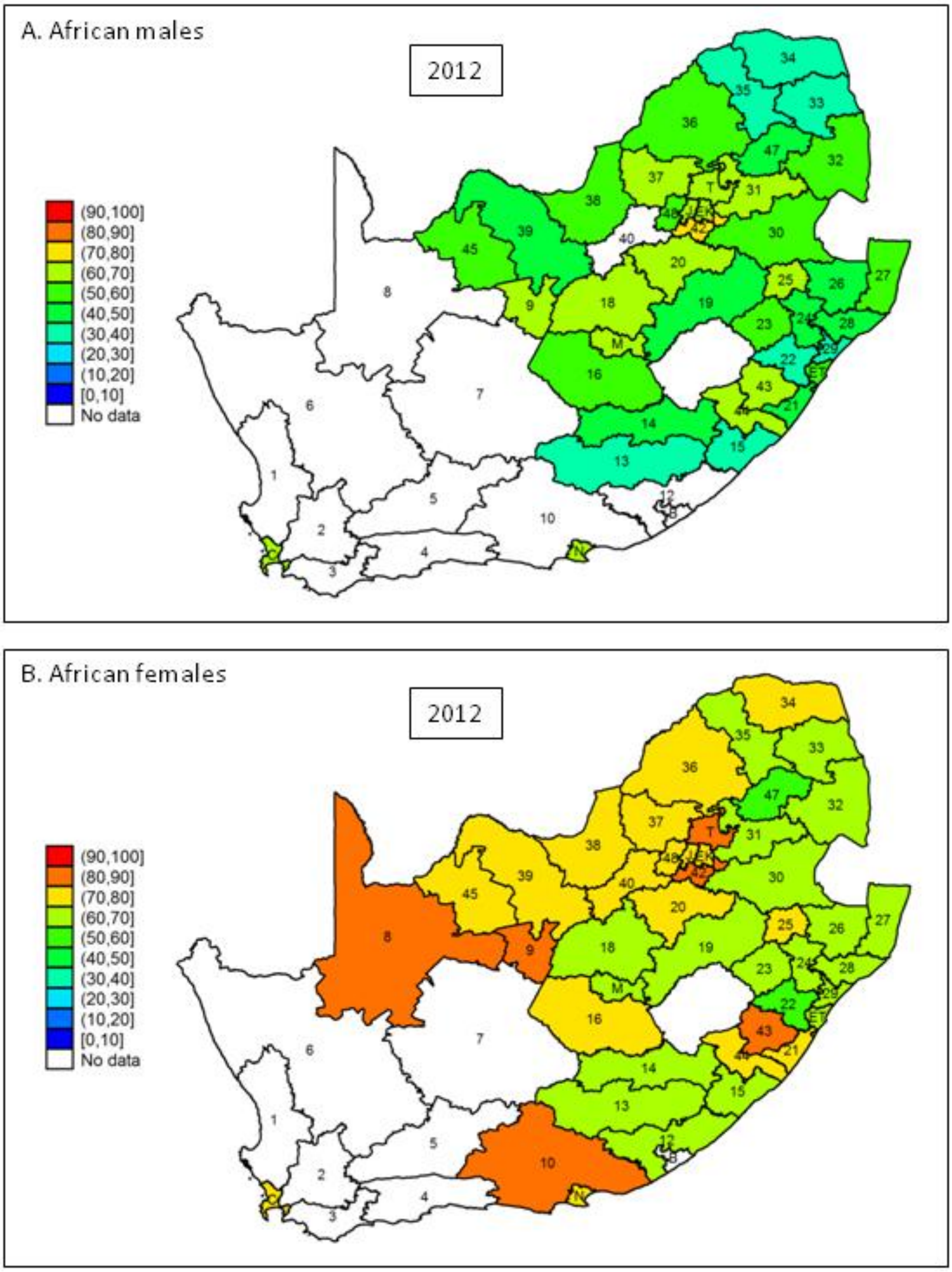

Figure A2: Cross-sectional data from 2012 showing the proportion of African men and women (15 years and older) individuals ever tested for HIV in each district. Figure (A) shows the districts for men and Figure (B) for women. Districts with less than 50 observations are not included. The data is weighted using the NIDS cross-sectional weights. See labels for Figure A1.
