# Supplementary material for: Changes in self-reported HIV testing during South Africa's 2010/2011 national testing campaign: gains and shortfalls
Source: J Int AIDS Soc. 2016 Apr 11;19(1):20658. doi: 10.7448/IAS.19.1.20658 (PMC4829657; doi:10.7448/IAS.19.1.20658)
Supplement: Changes in self-reported HIV testing during South Africa's 2010/2011 national testing campaign: gains and shortfalls [file JIAS-19-20658-s004.pdf]

Additional file 4

Table A3: Logistic regression models of factors associated with first-time HIV testing between 2010 and 2012 among black African populations living in urban informal areas and among black African women by reported pregnancy

Model	**Urban informal sample**<br>1<br>aOR [95%CI]	**African women pregnancy reported**<br>2<br>aOR [95%CI]	**African women no pregnancy**<br>3<br>aOR [95%CI]
**Female**	1.991**	na	na
	[1.007 - 3.940]	na	na
**Age**	1.071	0.972	1.062***
	[0.976 - 1.176]	[0.860 - 1.098]	[1.022 - 1.103]
**Age squared**	0.999	1.000	0.999***
	[0.998 - 1.000]	[0.998 - 1.001]	[0.999 - 0.999]
**Log real pc household income**	0.894	1.125	0.896
	[0.539 - 1.481]	[0.849 - 1.490]	[0.786 - 1.022]
**Years of education**	1.096*	1.078	1.091***
	[0.993 - 1.209]	[0.975 - 1.192]	[1.053 - 1.130]
**Currently enrolled in school**	1.213	0.848	0.434***
	[0.468 - 3.146]	[0.237 - 3.036]	[0.269 - 0.698]
**Unemployed (base = employed)**	1.095	2.304	1.091
	[0.350 - 3.419]	[0.574 - 9.249]	[0.705 - 1.687]
**Economically inactive (base = employed)**	1.453	1.511	1.037
	[0.457 - 4.621]	[0.606 - 3.769]	[0.735 - 1.465]
**Married/cohabitating**	0.983	1.001	1.226
	[0.500 - 1.934]	[0.479 - 2.095]	[0.897 - 1.674]
**Religion very important**	1.539**	1.359	1.138
	[1.043 - 2.272]	[0.825 - 2.238]	[0.940 - 1.377]
**Poor/fair health (base = good/excellent)**	0.887	1.365	0.739*
	[0.329 - 2.389]	[0.422 - 4.418]	[0.518 - 1.055]
**CESD 8 scale**	0.928**	0.952	1.006
	[0.875 - 0.984]	[0.882 - 1.027]	[0.978 - 1.036]
**Drinks alcohol**	1.118	1.891	1.111
	[0.649 - 1.927]	[0.716 - 4.995]	[0.690 - 1.789]
**Rural (base = urban formal)**	na	1.008	1.151
	na	[0.397 - 2.559]	[0.787 - 1.683]
**Urban Informal (base = urban formal)**	na	1.240	1.586
	na	[0.294 - 5.237]	[0.724 - 3.473]
**Days between interview**	1.000	1.001	0.999
	[0.997 - 1.003]	[0.997 - 1.006]	[0.998 - 1.001]
**Pregnant between waves**	1.909**	na	na
	[1.137 - 3.204]	na	na
**Controls for Province of residence**	No^	Yes	Yes
**Observations**	419	655	2,712

Notes: *** p<0.01, ** p<0.05, * p<0.1. 95% Confidence Intervals in brackets. Each column represents a separate logistic regression. The sample of interest is described in the header. The sample for Model 2 is all women who

reported having been pregnant during the study period. The sample for Model 3 is all women who did not report having been pregnant during the study period. All samples are restricted to those individuals surveyed both in 2010/2011 and 2012 who reported never having been tested for HIV in the former survey wave. As such, these regressions assess the determinants of first-time testing by 2012 among the sample of never testers in 2010/2011.

^Province controls not included due to insufficient sample size.
